# Treatment of Endometrial Cancer in Association with Pelvic Organ Prolapse

**DOI:** 10.1155/2017/1640614

**Published:** 2017-02-28

**Authors:** Asama Vanichtantikul, Ekkasit Tharavichitkul, Imjai Chitapanarux, Orawee Chinthakanan

**Affiliations:** ^1^Department of Obstetrics and Gynecology, Faculty of Medicine, Chiang Mai University, Chiang Mai, Thailand; ^2^Department of Radiology, Faculty of Medicine, Chiang Mai University, Chiang Mai, Thailand; ^3^Department of Obstetrics and Gynecology, Faculty of Medicine, Ramathibodi Hospital, Mahidol University, Bangkok, Thailand

## Abstract

*Background*. Uterine malignancy coexistent with pelvic organ prolapse (POP) is uncommon and standardized treatment is not established. The objective of this case study was to highlight the management of endometrial cancer in association with pelvic organ prolapse.* Case Report*. An 87-year-old woman presented with POP Stage IV combined with endometrioid adenocarcinoma of the uterus: clinical Stage IV B. She had multiple medical conditions including stroke, deep vein thrombosis, and pulmonary embolism. She was treated with radiotherapy and pessary was placed.* Conclusion*. Genital prolapse with abnormal uterine bleeding requires proper evaluation and management. Concurrent adenocarcinoma and POP can be a difficult clinical situation to treat, and optimum management is controversial.

## 1. Introduction

Pelvic organ prolapse (POP) is a common condition in the elderly. Effective treatments for POP include surgery. However, in the elderly population, the incidence of malignancy is increased and the risk peaks in the 75–84-year-old population. The incidence of endometrial cancer coexistent with POP varies from 0.2% to 1.2% [1].

The standard treatment for endometrial cancer is surgical treatment including hysterectomy with bilateral salpingo-oophorectomy (BSO) and pelvic and para-aortic lymphadenectomy [2]. In addition, vaginal hysterectomy is an alternative surgical treatment for endometrial cancer concomitant with POP particularly in those with morbid obesity or poor medical status [2, 3]. For adjuvant treatment, radiotherapy is generally used in women with intermediate risk of metastasis or recurrence; however, radiation alone could be considered in some circumstances [4].

To the best of our knowledge, there was no standard treatment for patients with endometrial cancer coexistent with POP. The objective of this case study was to highlight an alternative management of endometrial cancer in association with POP.

## 2. Case Report

**Figure 1:**
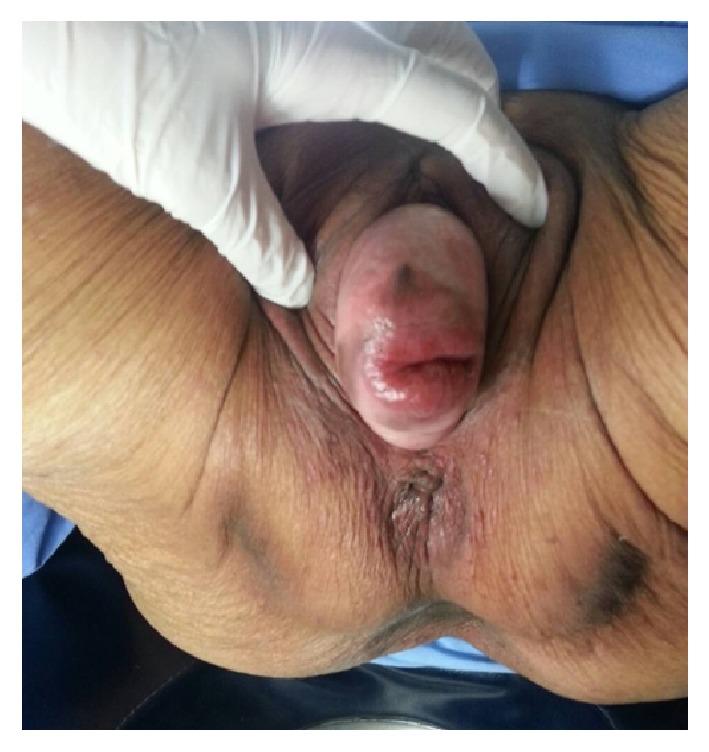
An 87-year-old woman with uterine prolapse Stage IV.

An 87-year-old Asian woman, para 7, was referred to our urogynecology clinic with a week of vaginal bleeding and untreated pelvic organ prolapse. She had no previous vaginal bleeding. The patient had a reducible prolapsed uterus for ten years. She denied any forms of treatment. She did not have any other complaints. This patient, ECOG performance status Grade 2, denied any hormonal replacement therapy or underlying medical conditions other than well-controlled hypertension. Her BMI was 17.6 kg/m^2^. On vaginal examination, it was found that there was POP Stage IV (POP-Q; Aa +3, Ba +5, C +6, Gh 8, Pb 2, TVL 7, Ap +3, Bp +3.5, D −1) (Figure 1), negative standing cough stress test with full bladder (reduced), normal sensation of perineum, weak and brief pelvic floor muscle strength, and good anal sphincter tone. In addition, there were no lesions in her vaginal wall and cervix, a slightly enlarged uterus, and there was no adnexal mass. Rectal examination showed no abnormalities. The endometrial sampling was performed with a curette at the outpatient department.

**Figure 2:**
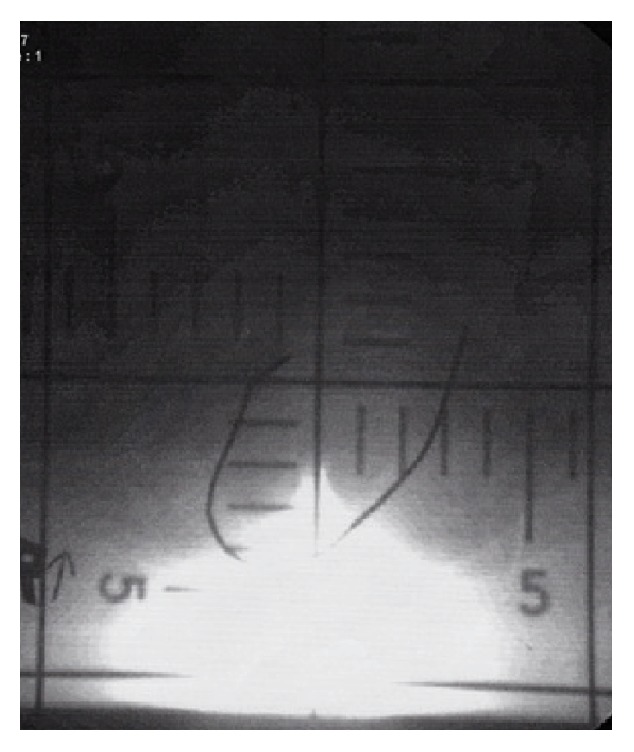
Localized small pelvic radiotherapy for endometrial cancer with prolapsed uterus.

Pathologic result from endometrial biopsy reported endometrioid adenocarcinoma, Grade 2. Imaging studies were evaluated. Her chest X-ray did not show any characteristic of metastasis. A computed tomography (CT) scan showed a uterine mass with bilateral ovarian and omental metastasis, clinical Stage IVB. The initial treatment plan was surgical management; however, she developed stroke and deep vein thrombosis of her left leg with pulmonary embolism. Secondary to her multiple medical comorbidities, her treatment plan was reconsidered and to include vaginal hysterectomy with BSO followed by radiation therapy, or radiotherapy alone. At the time, pelvic radiation either with uterine prolapse or with reduced uterus was considered. After discussion in our multidisciplinary oncology team of both possible treatment options, radiotherapy with a palliative aim on her primary tumor without reducing the prolapse was decided upon in order to avoid radiation side effects to adjacent organs. The radiation was performed 15 fractions (4,500 cGy) with external beam radiation 300 cGy per fraction (Figure 2). The simple small-field technique was used with hypofractionated regimen. After radiation was completed, her symptoms recovered and she developed Grade 2 dermatotoxicity (moist desquamation) that resolved within three months. She visited the radiation oncologist again at six months after radiation without symptoms. After discussion with her cousins, the best supportive care was designed to be further management. In addition, a ring with support pessary was placed for treating the POP.

## 3. Discussion

Uterine malignancy in pelvic organ prolapsed (POP) patients is uncommon, a range of 0.2%–1.2% risk of diagnosing uterine cancer after POP surgery [1]. In POP patients who present with postmenopausal bleeding, endometrial biopsy should be considered to exclude coexisting endometrial cancer [3].

Vaginal hysterectomy is an effective surgical treatment in POP patients. On the other hand, abdominal hysterectomy, complete surgical staging, was the mainstay of treatment for endometrial cancer patient. However, patients with endometrial cancer, particularly with Stage I disease, coexistent uterine prolapse, would best be treated with vaginal hysterectomy with BSO and/or peritoneal cytologic sample vaginally with vaginal repair [1, 3]. In advanced stage uterine cancer in POP patients, no ideal treatment has been proposed. After vaginal surgery, adjuvant treatment may play an important role. Nevertheless, the treatment options and postoperative adjuvant treatments are based on histologic cell type, stage, and risk factors of recurrence. In significantly medically compromised patients, surgical risk/benefit analysis should be evaluated against risk/benefit analysis of radiation therapy alone when guiding therapeutic decision making [2]. Recent publications showed that an average five-year disease-free survival rate in early stage endometrial cancer in medically compromised patients treated with primary radiotherapy and vaginal hysterectomy was 87%–95% and 90%, respectively [2, 4]. Various studies showed radiotherapy as a primary treatment is effective with a higher recurrence rate of 10–15% [4].

Treatment goals for this patient are a very important factor when making management decisions. Previous case reports have been published regarding the management in these cases. For curative aim, surgery should be performed to obtain accurate pathological details and radiation therapy is considered according to risks as normal condition [5].

For palliation, the aim of treatment was improvement of symptoms. The target of radiation was only the symptomatic lesion. Local irradiation to the problematic area with the smaller field was enough to control symptoms, in this case, regardless of the location of the lesion inside or outside of the pelvis.

POP with abnormal uterine bleeding requires careful evaluation. Uterine malignancy in POP patients is uncommon and standardized treatment is controversial. Radiotherapy is an alternative management.
